# Phosphorus Metabolic Disorder of Guizhou Semi-Fine Wool Sheep

**DOI:** 10.1371/journal.pone.0089472

**Published:** 2014-02-19

**Authors:** Xiaoyun Shen, Jinhua Zhang, Renduo Zhang

**Affiliations:** 1 Chongqing University of Science and Technology, Chongqing, China; 2 School of Environmental Science and Engineering, Guangdong Provincial Key Laboratory of Environmental Pollution Control and Remediation Technology, Sun Yat-sen University, Guangzhou, China; 3 Guizhou Normal University, Guiyang, China; 4 Office of Poverty Alleviation of Guizhou Province, Guiyang, China; 5 Guizhou Institute of Pratacultural, Guiyang, China; 6 Pratacultural Ecology Institute, Bijie University, Bijie, China; USGS National Wildlife Health Center, United States of America

## Abstract

Guizhou semi-fine wool sheep are affected by a disease, characterized by emaciation, lameness, stiffness in the gait, enlargement of the costochondral junctions, and abnormal curvature in the long bones. The objective of this study was to determine possible relationships between the disease and mineral deficiencies. Samples of tissue and blood were collected from affected and unaffected sheep. Samples of soil and forage were collected from affected and unaffected areas. The samples were used for biochemical analyses and mineral nutrient measurements. Results showed that phosphorus (P) concentrations in forage samples from affected areas were significantly lower than those from unaffected areas (*P <* 0.01) and the mean ratio of calcium (Ca) to P in the affected forage was 12:1. Meanwhile, P concentrations of blood, bone, tooth, and wool from the affected sheep were also significantly lower than those from the unaffected group (*P <* 0.01). Serum P levels of the affected animals were much lower than those of the unaffected ones, whereas serum alkaline phosphatase levels from the affected were significantly higher than those from the unaffected (*P <* 0.01). Inorganic P levels of the affected sheep were about half of those in the control group. Oral administration of disodium hydrogen phosphate prevented and cured the disease. The study clearly demonstrated that the disease of Guizhou semi-fine wool sheep was mainly caused by the P deficiency in forage, as a result of fenced pasture and animal habitat fragmentation.

## Introduction

Guizhou semi-fine sheep are vital to the production system of the Karst mountain areas of Southwest China. During the past 10 years, Guizhou semi-fine sheep have been affected by a disease, characterized by emaciation, lameness, enlargement of the costochondral junctions, and abnormal curvature in the long bones. Severe cases included permanent recumbency and eventual death. Based on preliminarily epidemiological and clinical data, this locally nutritional and metabolic disease may be associated with mineral imbalance for the animal.

The disorder has been observed throughout the years with peak incidence occurring between July and September and mainly occurred in ewes and lambs. In severe areas, 30% of sheep were affected and the mortality reached 15%. Similar syndromes have been reported in cattle [1], [2], water buffaloes [3], pigs [4], dogs [5], camels [6], and wild yaks [7], all of which are related to nutrition imbalance. However, there is no any available information about the disease affecting Guizhou semi-fine wool sheep. Therefore, the objective of this study was to determine the pathogeny of this disease and to establish possible relationships between the disease and mineral deficiencies.

## Materials and Methods

### Ethics statement

The sheep used in these experiments were cared as per outlined in the *Guide for the Care and Use of Animals in Agricultural Research and Teaching Consortium* [8]. Thirty affected and 30 unaffected sheep were slaughtered with electrical stunning then exsanguination, which was approved by the Institute of Zoology, Chinese Academy of Sciences, Institutional Animal Care and Use Committee (Project A0566). The experiments were conducted in the pasture of Bijie Comprehensive Experiment Station, Agriculture Research System, China. A permit was issued by the station authority (contact information: Ms. Lijuan Li, lzdxsxy@163.com, 8615086346733). The field studies did not involve endangered or protected species.

### Study area

The study area was located in a region adjoined by the provinces of Guizhou, Yunnan, and Sichuan (26°56'-27°47' N, 103°56'-104°51' E), with the average elevation 2100 m above the sea level, the annual precipitation 956 mm, and the average atmospheric temperature 9–11°C. The main grassland species include Puccinellia (*Chinam poensis ohuji*), Siberian Nitraria (*Nitraria sibirica pall*), Floriated astragalus (*Astragalus floridus*), Poly-branched astragals (*A. polycladus*), Falcate whin (*Oxytropis falcate*), Ewenki automomous banner (*Elymus nutans*), Common leymus (*Leymus secalinus*), and June grass (*Koeleria cristata*). Most of the plants are herbaceous and good resources for grazing animals.

### Epidemiological investigations

A detailed investigation on the epidemiology of the disease in the sheep was carried out in the affected area. Collected data included the history, incidence, character, and regularity of the disease, and the natural ecological conditions. We interviewed many local herdsmen who have been living in the area for many years, asking for background information on the disease. Data about the ecological and environmental conditions and their effects on the disease were obtained from local records and annual reports provided by the local government. Clinical signs were recorded by directly observing herd activities on the pasture.

### Sample collections

On July 1 of 2011, 60 Guizhou semi-fine wool sheep were selected for the following experiments, among which 30 were selected from 1650 sheep in affected pasture (26°58.5' N, 104°28.3' E). All the affected animals showed obvious clinical signs, including lameness, weakness, and enlargement of the costochondral junctions. Other 30 unaffected sheep were selected from Hezhang County of Guizhou Province (26°59.5' N, 104°52.3' E), where the disease had not been reported. Clinical examination showed that all of the unaffected animals were in good health, which was used as the control group.

Blood samples of the selected animals were obtained from the jugular vein using 1% sodium heparin as anticoagulant, and stored at –10°C for analysis of trace elements. Serum samples for biochemical analysis were taken in tubes without anticoagulant. The serum samples were separated by centrifugation (G: 10000–12000, time: 5–10 min, and plastic tube type: EF8977) and stored at –10°C in plastic vials. Wool samples were taken from the animal necks, washed, and degreased [9], and then kept on silica gel in a desiccator until analyses. After the affected and unaffected sheep were slaughtered, routinely post-mortem pathological examination was conducted by visually observing the tissues. Samples of ribs, hips, and teeth were collected from the animals to determine minerals in the tissues. The tissue samples were dried at 60–80°C for 48 h and stored on silica gel in a desiccator.

Samples of forage and soil were collected in July of 2011 in the affected areas (26°58.5' N, 104°28.3' E). Thirty forage samples were collected from randomly distributed locations, i.e., from 6 affected areas and 5 samples in each area. To reduce soil contamination, the herbage samples were cut 1–2 cm above the ground level [10], [11]. The forage samples were dried at 60–80°C for 48 h and ground to facilitate chemical analysis [12]. At the same locations, 30 soil samples were taken from the surface layer (0–30 cm), using a 30 mm diameter cylindrical corer. Each soil sample was composited by four soil cores collected at the site. The soil samples were dried at 60–80°C for 48 h and passed through a 2 mm sieve. Soil pH values were 6.8 to 7.2. Thirty forage samples were also collected from the unaffected grassland in Hezhang County.

### Biochemical examination

Lactate dehydrogenase (LDH), alkaline phosphatase (AKP), γ-glutamyl transferase (γ-GT), creatinine (CRT), calcium (Ca), inorganic phosphorus (IP), total protein (TP), albumin(Alb), and globulin (Glod) were determined using the serum samples and an automatic biochemical analyzer (OLYMPUS AU 640, Olympus Optical Co., Japan). Quality control serum (Shanghai Biochemical Co) was used to validate the blood biochemistry data. Serum protein electrophoretic studies were performed on cellulose acetate [13]. All serum biochemical values were measured at 20°C.

### Analysis of mineral contents

Concentrations of copper (Cu), iron (Fe), zinc (Zn), manganese (Mn), and Ca in samples of the animal tissues (blood, wool, ribs, hips, and teeth), soil, and forage were measured using a Perkin-Elmer AAS5000 atomic absorption spectrophotometer (Perkin-Elmer, Norwalk, Connecticut, USA). Molybdenum (Mo) content was determined using the flameless atomic absorption spectrophotometry (Perkin-Elmer 3030 graphite furnace with a Zeeman background correction). Fluorine (F) content was determined using ion chromatography (Metrohm MIC-7 advanced, Switzerland). Phosphorus was determined by spectrophotometry [12]. The accuracy of the analytical values was checked by reference to certified values of elements in the National Bureau of Standards (NBS) (bovine liver SRM 1577a).

### Treatment and prevention

In one severely affected area, 35 affected sheep were selected from 650 animals in the affected pasture for a treatment experiment. Among them, 10 affected sheep (5 males and 5 females) were given disodium hydrogen phosphate (Na_2_HPO_4_) orally at a dose of 50 g per animal and grazed on fenced pasture. The treatment was repeated once a week between July and September of 2011. The rest of the selected sheep grazed on the affected and fenced pasture without any treatment. Clinical signs were recorded by directly observing the sheep activities on the pasture. Specific attention was paid to signs of lameness, gait stiffness, and abnormal curvature in forelegs.

### Statistical analyses

Data were analyzed using the Statistical Package for the Social Sciences (SPSS, version 14.0, Inc., Chicago, Illinois, USA), and presented in the form of mean ± standard error (SE). Significant differences between groups were assessed using Student’s *t* test with least significant differences of 1% (*P* < 0.01) or 5% (*P* < 0.05).

## Results

### Epidemiology

The disorder mainly occurred in mature females and lambs of Guizhou semi-fine wool sheep throughout the year, with a peak incidence between July and September. Pregnant and post-partum females were most commonly affected by the disease. The clinical signs were less obvious in mature males. In severe areas, 30% of sheep were affected and the mortality reached 15%. Besides the symptoms described above, long bones of the affected sheep were broken frequently without apparent stress. However, body temperature, respiratory rate, and heart rate of the affected animals were normal.

### Autopsy findings

Visual autopsy examination showed that gross bone lesions of the affected lambs and adults were similar. Almost all bones, particularly the mandible, scapula, ilium, hip bone, and ribs, were affected. The affected bones were porous, brittle, light, susceptible to fracturing, and easier to be cut and sawn. The marrow cavity was enlarged and extended into the epiphysis, and the cortex was thin, spongy, and soft. Spontaneous fractures frequently occurred on ribs and pelves of the affected sheep. Enlargement of joints with apparent bowing of long bone and broadening of the epiphyses were typical. Many old enlarged scars were observed in ribs of affected adult females. Irregular ulcers were sometimes seen on the surface of joints. Kidneys were prominently enlarged and softened with a yellowish appearance, and livers were slightly swollen.

### Biochemical results

Serum AKP, LDH, and CRT of the affected sheep were significantly higher than those in the unaffected animals (*P <* 0.01), while IP levels were about half of those in the control group. The AKP values of the affected animals were two times higher than those of the unaffected sheep (Table 1). Likewise, concentrations of serum α-globulin and β-globulin of the affected sheep were significantly higher than those of the control group (*P <* 0.01). Serum α-globulin of the affected sheep was significantly lower than those in the control group (*P <* 0.01) (Table 2). There were no significant differences in other biochemical values between the affected and unaffected sheep.

**Table 1:** Biochemical parameters in serum samples of Guizhou semi-fine wool sheep.

Property	Affected	Unaffected
LDH^a^ (µmol/l)	5.86±1.38^b^	3.51±0.62
γ-GT (IU/l)	25.3±3.7	26.2±3.5
AKP (IU/l)	121±17.3^b^	56.2±8.7
CRT (µmol/l)	153±35^b^	116±21
Ca (mmol/l)	2.53±0.21	2.61±0.23
IP (mmol/l)	1.15±0.22^b^	2.28±0.22

a LDH  =  Lactate dehydrogenase; AKP  =  alkaline phosphatase; γ-GT  =  γ-glutamyl transferase; CRT =  creatinine; Ca  =  calcium; IP =  inorganic phosphorus.

b Results between the affected and unaffected Guizhou semi-fine wool sheep were significantly different (*P*<0.01).

**Table 2:** Serum protein concentrations (g/l) of Guizhou semi-fine wool sheep.

Items	Affected	Unaffected
Total protein	63.7±4.9	65.9±5.3
Albumin	45.3±3.7	46.7±3.6
α-Globulin	3.92±0.5^b^	2.82±0.7
β-Globulin	4.73±0.6^b^	3.12±0.6
γ-Globulin	9.83±0.7 b	13.3±1.7
A/G^a^	2.45±0.51	2.43±0.47

a A =  albumin;G =  globulin.

b Results between the affected and unaffected Guizhou semi-fine wool sheep were significantly different (*P*<0.01).

### Minerals

Concentrations of P in the soil and forage samples in the affected area were significantly lower than those in the unaffected area (*P <* 0.01) (Table 3). The P concentrations in the forage samples of the unaffected area were 5.4 times higher than those in the affected area. The mean Ca:P ratio in forage of the affected area was 12:1. Other values of the mineral elements were within the normal ranges. In addition, P concentrations in the blood and wool samples and in ribs, hips, and teeth from the affected animals were roughly half of those from the unaffected group (Tables 4 and 5).

**Table 3:** Mineral element concentrations (ppm) in soil and forage samples collected in the affected and unaffected areas.

Elements	Soil		Forage	
	Affected area	Unaffected area	Affected area	Unaffected area
Cu	16.7±2.9	16.9±2.7	6.9±2.3	6.3±2.7
Mo	1.33±0.37	1.28±0.38	1.16±0.37	1.13±0.38
Fe	4527±371	4332±323	376±31	382±37
Zn	28.7±4.3	28.2±5.1	5.5±1.2	5.3±1.7
Mn	55.7±11.5	53.8±11.2	13.2±3.9	13.3±3.6
Ca	12278±457	12719±419	2866±217	2613±192
P	31.9±6.5^a^	56.5±6.7	239±13^a^	1279±31
F	25.78±5.8	25.23±6.3	21.5±4.9	22.5±5.3
Ca: P	385:1	225:1	12:1	2:1

a Results between the affected and unaffected areas were significantly different (*P*<0.01).

**Table 4:** Mineral element concentrations in the whole heparinised blood and wool samples of the Guizhou semi-fine wool sheep (The unit for F is in ppb and others in ppm).

Elements	Blood		Wool	
	Affected	Unaffected	Affected	Unaffected
Cu	0.75±0.23	0.76±0.25	5.18±1.23	5.15±1.31
Mo	0.36±0.06	0.37±0.07	0.31±0.05	0.33±0.09
Fe	512.3±22.9	513.6±23.8	333.2±21.6	337.5±17.5
Zn	15.3±2.9	15.7±2.7	87.5±4.8	89.3±3.8
Mn	0.51±0.15	0.58±0.17	5.77±1.16	5.73±1.17
Ca	127.0±13.7	127.3±11.5	1019±41	1097±67
P	253.0±22.4^a^	368.0±32.4	81.9±13.7^a^	157.7±15.7
F	18.7±5.4	19.1±6.1	18.9±4.7	19.7±5.7

a Results between the affected and unaffected Guizhou semi-fine wool sheep were significantly different (*P*<0.01).

**Table 5:** Mineral element concentrations in bones and teeth of Guizhou semi-fine wool sheep (The unit for Ca and P is in g/kg dry sample and others in ppm).

Elements	Rib		Hip		Teeth	
	Affected	Unaffected	Affected	Unaffected	Affected	Unaffected
Cu	7.93±2.2	7.64±2.3	5.78±1.56	5.69±1.37	4.82±0.73	4.87±0.76
Mo	1.37±0.61	1.35±0.51	2.57±0.51	2.53±0.61	2.35±0.57	2.48±0.53
Fe	179.9±11.9	173.2±12.7	177.7±14.8	175.3±15.7	157.5±11.1	153.7±12.5
Zn	113.7±11.9	112.7±12.9	98.3±7.8	97.7±7.3	92.8±5.7	91.9±5.1
Mn	6.67±1.31	6.59±1.49	4.53±1.21	4.57±1.13	6.17±0.53	6.13±0.58
Ca	131.9±12.1	137.5±12.2	127.6±17.5	125.6±23.1	177.3±22.6	173.8±23.7
P	37.3±5.7^a^	75.7±12.6	35.1±3.1^a^	78.3±11.6	32.3±6.1^a^	77.6±7.5
F	57.3±13.7	55.7±12.6	65.1±13.1	63.3±11.6	72.3±16.1	77.6±7.5

a Results between affected and unaffected the Guizhou semi-fine wool sheep were significantly different (*P*<0.01).

### Treatment and prevention

Animals treated with Na_2_HPO_4_ recovered gradually within 10 to 20 d. Generally, appetite improved quickly and signs of lameness in most animals improved within 5 to 10 d after the treatment. However, foreleg deformation recovered slowly and required prolonged treatment. Females and lambs were more vulnerable than males in the treated and untreated sheep. The 10 treated sheep totally survived. Among the 25 of untreated animals, 5 males survived, 2 of 10 females and 2 of 10 lambs eventually died. It was noteworthy that 2 of 10 untreated females died and none of 5 treated females died, while none of untreated and treated males died.

## Discussion

Several mineral nutrition related diseases of sheep have been studied in the literature. Huang (2001) and Huang and Chen (2002) reported the pathogenesis of Tibet sheep and goats because of sulfur (S) and Cu deficiency in forage in Gansu Province of China [14], [15]. Shen (2011) reported a disease of semi-fine wool sheep in Guizhou Province, which was related to S deficiency caused by high Fe in forage. The main signs of the disease included wool-eating, emaciation, losing appetite, pica, and weight loss [16]. Yuan et al. (2011) reported another disease of semi-fine wool sheep in Guizhou Province, which was caused by Cu deficiency mainly due to high S and Mo content in forage [17]. Compared with the diseases above, the disease in this study occurred in a different region, i.e., the adjoined region of Guizhou, Yunnan, and Sichuan Provinces, with different characteristics, and different nutrition deficiency problems. Therefore, it was the first time to report this disease of Guizhou semi-fine wool sheep.

Preliminary epidemiological and clinical observations indicated that Guizhou semi-fine wool sheep suffered a nutritional and metabolic disease associated with P deficiency. Our experimental results showed that the P concentrations in the soil and forage in the affected areas were significantly lower than the unaffected areas. In addition, P levels in serum, wool, bones, and teeth from the affected sheep were markedly lower, and serum AKP level was significantly higher than those of the unaffected animals. The result was consistent with the response criteria in P deficiency disease of wildlife yaks and Bactrian camels [6], [7], [18]. The oral supplement of Na_2_HPO_4_ appeared to cure the disease successfully. The above results clearly demonstrated that the disorder problem of Guizhou semi-fine wool sheep was related to the P deficiency in forage, which was attributable to the current herding practices as discussed below.

The local herd practices have a great impact on nutrition imbalance for grazing animals. In 1990s, the pasture and livestock were allocated to individual families in an attempt to improve the local herdsmen’s life and productivity. Animals grazed in fixed and limited areas within fenced pasture. As a result, the fenced pasture and habitat fragmentation might create nutrition imbalance problems for animals. Nutrient contents in the soil and forage are spatially distributed. If animals graze in an extensive area, they have chances to graze in poor as well as rich nutrition areas. Therefore, the nutrition imbalance problem is minimal [19]. In the present study, P concentrations in the soil and forage from the affected areas were significantly lower than those in the unaffected areas. Within the fenced pasture, the sheep grazed in the same pasture with P deficiency during the year. As a result, Guizhou semi-fine wool sheep suffered the disease related to the P deficiency.

For many grass species, the period with relatively high P concentrations (>0.3%) available to grazing animals is pretty short [20]–[22]. In most years, mature forage contains P <0.15% [4], [23]. In general, the sufficient P levels for ruminants are >0.005% in soils and >0.3% in forage [3], [24]. However, in the present study, the P levels of the soil and forage in the affected areas were 0.0031% and 0.024%, respectively, which were much lower than the sufficient levels.

Among the factors influencing Ca and P utilization metabolism, a Ca:P ratio of 1:1 to 2:1 is usually recommended for proper utilization of the elements by animals [2], [25]. Dietary Ca:P ratios <1:1 or >7:1 should adversely affect growth and feed efficiency of animals [21], [26]. In our study, the mean Ca:P ratio in forage from the unaffected area was 2:1. However, the mean Ca:P ratio in forage from the affected area was 12:1, which should have a negative impact on the Ca and P metabolism of the semi-fine wool sheep in the area. To prevent P deficiency in grazing livestock, oral supplement of bone meal, phosphate and mineral mixtures is recommended [1], [4].

A number of response criteria have been used to evaluate the P status of animals, including serum levels of P, Ca, and alkaline phosphatase (AKP). Previous research suggests that bone criteria are more sensitive to P than to other elements. A marked hypophosphataemia is also a good indicator of a severe P deficiency, even if serum levels of Ca are unaffected. Phosphorus levels of blood samples are not a good indicator for the P status because P levels can be normal for a long period after animals have been exposed to serious P deficiency [25], [27].

Phosphorus deficiency disease should be differentiated from chronic fluorosis in mature animals. The typical characteristic of fluorine toxicity includes mottling and pitting of teeth and enlargements on the shafts of long bones. In this study, the fluorine concentrations in soil and forage were lower than the critical values of 30–40 ppm [28]. The fluorine concentrations in bones, tissues, blood, and wool were within the normal range. Therefore, the disease of Guizhou semi-fine wool sheep was not related to fluorosis. A general opinion is that sheep rarely suffer clinically from P deficiency [29]. However, mature females and lambs are more susceptible to P deficiency than mature males.
